# Principles of Stress-Strength Modelling of the Highly Thermally Loaded Materials—The Influence of an Effect of Strength Differential on the Material Effort

**DOI:** 10.3390/ma14237449

**Published:** 2021-12-04

**Authors:** Tomasz Ochrymiuk, Waldemar Dudda, Marcin Froissart, Janusz Badur

**Affiliations:** 1 Institute of Fluid-Flow Machinery, Polish Academy of Sciences, 14 Fiszera Street, 80-231 Gdańsk, Poland; mfroissart@imp.gda.pl (M.F.); jb@imp.gda.pl (J.B.); 2 Faculty of Technical Sciences, University of Warmia and Mazury in Olsztyn, 11E Oczapowskiego Street, 10-736 Olsztyn, Poland; dudda@uwm.edu.pl

**Keywords:** Huber–Mises–Hencky, Burzyński, effort hypotheses, strength differential (SD), St12T steel

## Abstract

This paper presents an improvement in the Huber–Mises–Hencky (HMH) material effort hypothesis proposed by Burzyński. Unlike the HMH hypothesis, it differentiates the plastic effort between compression and tensile load states, and links shear with tensile limit. Furthermore, it considers the fact that construction materials do not have infinite resistance in the pure tensile hydrostatic load state, which was proved by the static load experiment performed on St12T heat-resistant steel. The asymmetry between tensile and compressive loads is captured by the elastic region asymmetry coefficient ϰ, which was established by experiment for St12T steel in the temperature range between 20 °C and 800 °C.

## 1. Introduction

Demonstrating thermal stresses and strains is generally a complex and challenging task. Typically, if the acceleration time is long enough transient thermal gradients are significantly reduced keeping thermal stresses well below the yield strength. On the other hand, in the case of steep and fluctuated loads containing rapid cooling–heating cycles, the thermal stresses can become much higher than yield strength inducing plastic deformation. This is particularly harmful for areas with a high stress concentration.

During the design process of a highly thermally loaded components such as combustion chambers, special attention should be paid to stress–strength modelling. It is even more important in cases such as the use of modern experimental materials, such as sintered metals or orthotropic reinforced ceramics, because of their limited current application. According to commonly accepted procedures in the gas turbine industry, it is good practice to apply additional non-standard thermal-strength models. The reasons for this approach are as follows: the first obvious purpose is the verification of standard models that have been widely used for years. The second, less obvious reason is to improve existing methods via the detection of phenomena which do not exist in classical materials. In such a case, classical tools need to be upgraded or replaced to follow the progress in materials engineering. The fluid–solid interaction approach is particularly important in improving the accuracy of load state prediction. The following paper proposes a novel model of the phenomena, with an emphasis on viewing strength variability as a function of material load (effort). This approach was used to model the stress and deformation in turbine components, and is successfully tailored to describe the physical phenomena present in the wet combustion liner designed in the project.

Heat-resistant materials such as chromium steel, are the subject of numerous testing campaigns aimed at improving the durability of gas turbine combustion chambers [1]. That state has lasted for nearly 80 years to date, and is going to be extended into the future. The growing market share of sustainable energy generators in the power system requires flexible steam turbines that are capable of compensating for that variability. That inevitably leads to the accumulation of many low-cycle fatigue cycles in steam turbine units, and is associated with the wider range of operating conditions from idle to peak load, where steam temperature can exceed 600 °C.

The most challenging thermal conditions occurring within the constructions used in conventional power plants are caused by [2] thermal gradients induced by acceleration and deceleration, tight joints restraining thermal expansion, and mechanical and thermal low-cycle fatigue [3,4]. Steadily growing performance requirements of power plants, in conjunction with more frequent low-cycle fatigue cycles, can lead to the early life consumption of turbine components. Following that trend, research institutes search for stronger and cheaper materials for critical power plant elements such as rotors and boilers to improve their life and robustness [5].

The main objective of the presented paper is the description of stress and strain states in highly thermally loaded materials using both the Huber–Mises–Hencky (HMH) and Burzyński effort criteria [6,7,8,9,10,11,12,13,14,15]. The HMH approach is certainly the most commonly employed approach to capture effort evolvement. However, the HMH method should be limited to the materials with no tensile–compressive strength difference ($k_{c}=k_{t}$) and no shearness difference ($k_{s}=k_{t}/2$). In any other case of heat-resistant steels, for which $\varkappa =k_{c}/k_{t}=1.12÷1.24$, the classical Huber–Mises–Hencky hypothesis is inadequate.

As an extension of the HMH criterion, in the present paper Burzyński’s extended hypothesis has been employed to capture the asymmetry between compressive and tensile load regimes, as well as the shearness ($k_{s}\neq k_{t}/2$). This extension is also based on an additional aspect of elastic energy, known in the literature as “the thermal energy”. Numerical simulations include an asymmetry parameter derived from the experiment run in several thermal conditions, which is a novel approach in such models [16,17]. This unique method is barely present in the literature and includes an additional Burzyński component based on the energy of volumetric deformation. Such modification of the HMH hypothesis (from a one-parameter to a three-parameter model) makes it more robust and applicable to a wider selection of construction materials. The intention of the authors is to combine effort criterions used for elasto-plastic and elasto-brittle materials into one universal approach, considering the impact of the second principal stress $\sigma_{2}$ (that is not included in the Tresca and Mohr hypotheses). A key conclusion to emphasize within the power plant designer community is that, in some cases, the HMH hypothesis overestimates the safety margin. On the other hand, Burzyński’s hypothesis makes it more realistic and applicable for thermally loaded structures.

Generally speaking, the aim of this paper is two-fold. Firstly, it elaborates a new model of the effort limit for heat-resistive steels. Secondly, it shows how this model behaves within the elevated temperature range up to 800 °C.

Currently, due to the industrial attention paid to ceramic materials, this is a subject of interest among the scientific community [18,19]. The for this reason is that this group of materials has excellent functional properties, i.e., a heat and thermal gradient resistance up to ultra-high temperatures (1100 °C). As a result of that, ceramic materials have many potential applications in aerospace engineering, e.g., turbine blades or combustion chambers [20,21].

## 2. The Material Effort Description by the Energy Approach

It is well known that a “father” of the notion of “material effort” is James Clerk Maxwell. In general, this notion is different from the notion of “strength of material” and only within the field of HMH models do both notions coincide (strength is just a critical effort). Note that Maxwell introduced his concept within the frame of energy-based approaches. In particular, in 1856, in a letter to William Thomson he introduced the concept of “distortional energy” as a part of elastic strain energy, that is, the best candidate to measure material effort [2,10]. Therefore, in our numerous papers on this subject, we differentiate two notions: “effort hypothesis”, and “strength hypothesis”. Note that the notion of “material effort” is fully consistent with the Maxwell energy approach. In the complete mathematical form, this energy-based approach was initiated by Beltrami (1885) in the form [9]: Φ≤K, where Φ is some elastic deformation energy (precisely, the volumetric density of energy) describing a state of material effort, and K is a critical value of this energy. Beltrami first found that critical energy K depends on the uniaxial yield $k_{t}$ or torsion $k_{s}$. This approach also opened up the possibility of using many other forms of experimental data such as: Vigers hardness, Sharpy energy, toughness critical energy, cleavage energy, and so on (see Orłowski et al. [22,23]).

Typical energy types of the effort hypothesis can be classified as [2,17,24,25]:

- Beltrami (1885):

(1) $$\Phi =\sigma_{ij}\varepsilon_{ji}=\Phi \left(\sigma_{ij},\;\theta \right)=\Phi \left(\varepsilon_{ij},\;\eta \right)\leq K$$

- Huber (1904):

(2) $$\Phi ={\;\Phi }_{v}+\Phi_{f}\leq K\left(\mathrm{tension})\;\mathrm{and}\;\Phi_{f}\leq K\;(\mathrm{compression}\right)$$

- Mises (1914) and Hencky (1924):

(3) $$\Phi_{f}\leq K$$

- Schleicher (1926):

(4) $$\Phi ={\;\Phi }_{v}\left(\nu^{*}\right)+\Phi_{f}\left(\nu^{*}\right)\leq K$$

- Burzyński (1928):

(5) $$\Phi =\eta_{v}\Phi_{v}+\Phi_{f}\leq K$$

(6) $$\eta_{v}=\omega +\delta /p$$

- Zawadzki (1956):

(7) $$\Phi ={\;\Phi }_{v}+\Phi_{f}+\Phi_{th}\leq K$$

- Pęcherski (2011):

(8) $$\Phi =\eta_{v}\Phi_{v}+\eta_{f}\Phi_{f}\leq K$$

(9) $$\eta_{f}=1+\alpha \left[1-e^{-\beta (1+\cos\left(3\theta \right)}\right]$$

In the list above, several parameters appear: the distortional strain energy $\Phi_{f}$, the volumetric strain energy $\Phi_{v}$, and the thermal strain energy $\Phi_{th}$. These classical energies are corrected by the influence functions $\eta_{v}$ and $\eta_{f}$ which are a kind of factors introducing new parameters into the criterion [12,16,26,27].

In this approach, it is important to know the value of critical energy:

(10) $$K=\sqrt{2E\;k_{t}}$$

where 0.01<K<0.30 (as an example) for $k_{t}=700\;$MPa, E=2.1 GPa, and K=0.23 MJ.

From the perspective of required experimental parameters, the above criteria can be classified as: one-parameter (Beltrami, Huber, HMH), two-parameters (Schleicher), three-parameters (Burzyński, Zawadzki), and five-parameters (Pęcherski). Note that the energetic approach is dedicated to the question of multi-parameter criteria in cases where parameters represent physically different phenomena and dimensionalities. For instance, “limiting cleavage” is, from the very beginning, given in terms of [Joule] not in terms of [MPa]. Another example is “limiting hardness”, which is given in Brinell or Vickers scale units. The other candidates in the multi-parameter effort hypothesis are: brittleness, adhesiveness, gumminess, chewiness, and resilience [13,15]. If robust scientific tools for measuring these limiting parameters can be obtained, then a general framework for finding the principles of energy-like effort modeling, even with 15 parameters, is possible. Therefore, our paper investigates this question from the very beginning.

### 2.1. The Huber Material Effort

In 1903 Huber proposed an energetic measure of equivalent stress that is based on the energy of elastic deformation [9]:

(11) $$U=\int^{\;}\Phi dv=\int^{\;}\sigma_{ij}\varepsilon_{ij}dv\;\equiv \int^{\;}\sigma_{eq}\varepsilon_{eq}dv$$

In terms of stresses:

(12) $$U=\int^{\;}\Phi dv=\int^{\;}\frac{1}{2}\;C_{ijkl}\sigma_{ij}\sigma_{kl}dv\;\equiv \int^{\;}\frac{1}{2E}\sigma_{eq}\sigma_{eq}dv$$

Note that, originally, his equation for energy density was expressed by three main strains [9], which is quite easy to decompose into distortional and volumetric parts:

(13) $$\Phi =\frac{1}{2}H{\left(\lambda_{1}+\lambda_{2}+\lambda_{3}\right)}^{2}+\frac{1}{3}G\left[{\left(\lambda_{1}-\lambda_{2}\right)}^{2}+{\left(\lambda_{2}-\lambda_{3}\right)}^{2}+{\left(\lambda_{3}-\lambda_{1}\right)}^{2}\right]=\Phi_{v}+\Phi_{f}$$

where H=E/3(1−2ν) and G=E/2(1+ν) are the Helmholtz and Kirchhoff coefficients, respectively. It is important that energy can be split into volumetric and distortional (shear-like) components: $\Phi_{v}+\Phi_{f}$**,** where shear energy is:

(14) $$\Phi_{f}=\frac{1}{12G}\left[{\left(\sigma_{1}-\sigma_{2}\right)}^{2}+{\left(\sigma_{2}-\sigma_{3}\right)}^{2}+{\left(\sigma_{3}-\sigma_{1}\right)}^{2}\right]=\;=\frac{1}{2G}J_{2s}=\frac{1}{3G}\;q^{2}=\frac{3}{4G}\;\tau_{n}^{\;2}=\frac{1}{6G}\;\sigma_{HMH}^{\;2}$$

Three main stresses appear, q (distortion stress), $\tau_{n}$ (octeadric stress), and $\sigma_{HMH}$ (Huber–Mises–Hencky stress) that are well known from the literature [17,24,25]. This is alongside another frequently used quantity, the second invariant of the deviatoric stress tensor $J_{2s}$.

Here, a key stress invariant appears for the first time [11,13,14]:

(15) $$\sigma_{HMH}=\sqrt{3J_{2s}}=\sqrt{\frac{3}{2}\;s\cdot s}$$

(16) $$q=\sqrt{2J_{2s}}=\sqrt{s\cdot s}$$

(17) $$\tau_{n}=\sqrt{\frac{2}{3}J_{2s}}=\frac{1}{\sqrt{3}}\sqrt{s\cdot s}$$

where $s=\sigma -I_{\sigma }I\;$is the stress deviator. In the Western European literature, there are different notations for the relationship between invariants, $\sqrt{3J_{2s}}$, $\sqrt{2J_{2s}}$, or $\sqrt{\frac{2}{3}J_{2s}}\;$:

(18) $$\sigma_{i}=\sigma_{HMH}=\sqrt{\frac{3}{2}}\;q=\frac{3}{\sqrt{2}}\;\tau_{n}$$

(19) $$\sigma_{HMH}>q>\tau_{n}$$

(20) $$\Phi_{f}=\frac{1+\nu }{E}\;J_{2s}=\frac{1+\nu }{3E}\;\sigma_{HMH}^{2}$$

(21) $$G=\frac{E}{2\left(1+\nu \right)}$$

or in terms of the principal stresses:

(22) $$J_{2s}=\frac{3}{2}\tau_{oct}^{2}=\frac{1}{2}q^{2}=\frac{1}{3}\sigma_{i}^{2}$$

(23) $$\sigma_{i}=\sigma_{HMH}=\sqrt{3J_{2s}}=\frac{1}{\sqrt{2}}\sqrt{{\left(\sigma_{1}-\sigma_{2}\right)}^{2}+{\left(\sigma_{2}-\sigma_{3}\right)}^{2}+{\left(\sigma_{3}-\sigma_{1}\right)}^{2}}$$

(24) $$q=\sqrt{2J_{2s}}=\frac{1}{\sqrt{3}}\sqrt{{\left(\sigma_{1}-\sigma_{2}\right)}^{2}+{\left(\sigma_{2}-\sigma_{3}\right)}^{2}+{\left(\sigma_{3}-\sigma_{1}\right)}^{2}}$$

(25) $$\tau_{oct}=\tau_{n}=\sqrt{\frac{2}{3}J_{2s}}=\frac{1}{3}\sqrt{{\left(\sigma_{1}-\sigma_{2}\right)}^{2}+{\left(\sigma_{2}-\sigma_{3}\right)}^{2}+{\left(\sigma_{3}-\sigma_{1}\right)}^{2}}$$

The names of these invariants are: “stress intensity”, “Prager intensity”, and “the octahedral invariant”, respectively [2,3,17,25].

### 2.2. An Extended Burzyński Material Effort

The necessity of extension of classical HMH life limiting hypothesis was proven by the heat resistant steels experiment. It indicated that the limit in uniaxial tension is different to the limit in uniaxial compression, so the one-parameter HMH hypothesis must be replaced with a two-parameter one. In general, according to the experimental data [1,4,28,29], there are several possible limiting parameters even in the single uniaxial probe: elastic limit, yield stress, maximum strength limit, and failure limit. In order to express the fact of generality within the energy approach, let us denote all of these limits collectively by $k_{t}$. However, in the next section, focusing our attention only on plasticity, we replaced instances of $k_{t}$ with the letter $\sigma_{e}^{t}$. Burzyński indicates [6,7] that seven different load regimes should be tested: uniaxial tensile limit $k_{t}$, uniaxial compression limit $k_{c}$, torsional limit $k_{s}$, bi-axial tensile $k_{tt}$, bi-axial compression $k_{cc}$, three-axial tensile $k_{ttt}$, and three-axial compression $k_{ccc}$. Note that, usually, four basic limits of the effort state $k_{t}$ are taken into consideration: elastic $R_{e}$, plastic $R_{pl}$, extremal $R_{m}$, and rupture $R_{z}$. The main motivation for extension of the one-parameter HMH hypothesis is the well-known Duguet–Mohr hypothesis for materials with $k_{c}>k_{t}$:

(26) $${\left(\sigma_{1}-\sigma_{3}\right)}^{2}+\left(k_{c}-k_{t}\right)\left(\sigma_{1}+\sigma_{3}\right)=k_{c}k_{t}$$

In 1927, using the achievement of his supervisor Huber as a starting point, young Burzyński proposed a two-parameter extension of the Huber effort model [7]. Now, let us revalorize his line of reasoning by adding the thermal energy term $\Phi_{th}$, which derives from the thermal expansion of a solid material:

(27) $$W=\Phi_{f}+\eta_{\nu }\Phi_{v}+\Phi_{th}\leq K$$

where W is a measurement of effort (in terms of energy not stress), which is a quasi-linear composition of $\Phi_{f},\;\Phi_{\upsilon }$ and the parameter $\eta_{\nu }$, which is a function of pressure p and two constants ω,δ:

(28) $$\eta_{\nu }=\omega +\frac{\delta }{3p}$$

Equation (29) was created by inserting Equation (28) into Equation (27):

(29) $$\Phi_{f}+\left(\omega +\frac{\delta }{3p}\right)\Phi_{\upsilon }+\Phi_{th}=K$$

which can be written in an extensive form as:

(30) $$\frac{1}{6G}\;\sigma_{HMH}^{\;2}+\omega \left[\frac{1-2\nu }{12G\left(1+\nu \right)}{\left(3p\right)}^{2}+\frac{1}{2}\alpha \Delta T\left(3p\right)\right]+\delta \left[\frac{1-2\nu }{12G\left(1+\nu \right)}\left(3p\right)+\frac{1}{2}\alpha \Delta T\right]=K$$

Next, substituting:

(31) $$\omega \frac{1-2\nu }{1+v}=\frac{1-2\tilde{\nu }}{1+\tilde{\nu }}$$

(32) $$12GK=\frac{3k_{c}k_{t}}{1+\tilde{\nu }}$$

(33) $$\delta \frac{1-2\nu }{1+\nu }=\frac{3\left(k_{c}-k_{t}\right)}{1+\tilde{\nu }}$$

after multiplication by $12G\left(1+\tilde{\nu }\right)$ the following form is obtained:

(34) $$\frac{2}{3}\left(1+\tilde{\nu }\right)\sigma_{HMH}^{\;2}+3\left(1-2\tilde{\nu }\right)p^{2}+3\left(k_{c}-k_{t}\right)p+\;+6\omega G\left(1+\tilde{\nu }\right)\alpha \Delta T\;p+2\delta G\left(1+\tilde{\nu }\right)\alpha \Delta T=k_{c}k_{t}$$

or, in brief:

(35) $$\frac{2}{3}\left(1+\tilde{\nu }\right)\sigma_{HMH}^{\;2}+3\left(1-2\tilde{\nu }\right)p^{2}+3\left(k_{c}-k_{t}+ak_{t}\right)p+b=k_{c}k_{t}$$

where:

(36) $$3ak_{t}p+b\equiv 6\omega G\left(1+\tilde{\nu }\right)\alpha \Delta T\;p+2\delta G\left(1+\tilde{\nu }\right)\alpha \Delta T$$

This means that a and  b are:

(37) $$a=2\frac{G}{k_{t}}\omega \left(1+\tilde{\nu }\right)\alpha \Delta T\;\;\;$$

(38) $$b=2\delta G\left(1+\tilde{\nu }\right)\alpha \Delta T$$

In terms of $k_{t},\;\varkappa ,\;\mathrm{and}\;\tilde{\nu }$ our extended Burzyński hypothesis takes the following form:

(39) $$\frac{2}{3}\left(1+\tilde{\nu }\right)\sigma_{HMH}^{\;2}+3\left(1-2\tilde{\nu }\right)p^{2}+3k_{t}\left(\varkappa -1+a\right)p+b=k_{c}k_{t}$$

Another form can be obtained when the part:

(40) $$\frac{2}{3}\left(1+\tilde{\nu }\right)\sigma_{HMH}^{\;2}+3\left(1-2\tilde{\nu }\right)p^{2}$$

This is expressed as:

(41) $$\sigma_{1}^{2}+\sigma_{2}^{2}+\sigma_{3}^{3}-2\tilde{\nu }\left(\sigma_{1}\sigma_{2}+\sigma_{2}\sigma_{3}+\sigma_{3}\sigma_{1}\right)$$

Then, the extended (validated) Burzyński criterion has the form:

(42) $$\sigma_{1}^{2}+\sigma_{2}^{2}+\sigma_{3}^{3}-2\tilde{\nu }\left(\sigma_{1}\sigma_{2}+\sigma_{2}\sigma_{3}+\sigma_{3}\sigma_{1}\right)+\;+k_{t}\left(\varkappa -1+a\right)\left(\sigma_{1}+\sigma_{2}+\sigma_{3}\right)+b=k_{c}k_{t}$$

If a  and b →0, then the original Burzyński formulae is obtained [7]. If a=0 and $\tilde{\nu }=0.5$, then the other Burzyński form arises [7]. Finally, when b=0 (meaning ϰ=1) a traditional HMH condition is formed.

## 3. Huber–Mises–Hencky and Burzyński Equivalent Stress

Numerical modelling in the design process needs to be applied for different structural elements to capture the state of material effort at every critical point in a structure. Usually, the design process assumes certain stress margins to ensure the robustness and safety of the final product. Strictly speaking, designers must always keep component effort below the strength limit. Typically for steels, when $k_{t}=k_{c}$, we use the well-established one-parameter Huber–Mises–Hencky (HMH) hypothesis to describe the effort of the given feature. In such a case, the equivalent stress could be written using the following relationships:

(43) $$\sigma_{HMH}=\sqrt{3J_{2s}}=\sqrt{\frac{3}{2}\;s\cdot s}$$

(44) $$\sigma_{HMH}=\frac{1}{\sqrt{2}}\sqrt{{\left(\sigma_{1}-\sigma_{2}\right)}^{2}+{\left(\sigma_{2}-\sigma_{3}\right)}^{2}+{\left(\sigma_{3}-\sigma_{1}\right)}^{2}}$$

(45) $$\sigma_{HMH}=\sqrt{\frac{1}{2}\left[{\left(\sigma_{xx}-\sigma_{yy}\right)}^{2}+{\left(\sigma_{yy}-\sigma_{zz}\right)}^{2}+{\left(\sigma_{zz}-\sigma_{xx}\right)}^{2}\right]+3\left(\sigma_{xy}^{2}+\sigma_{yz}^{2}+\sigma_{zx}^{2}\right)}$$

where: $J_{2s}$ is the second invariant of the stress deviator; s is the stress deviator; $\sigma_{1},\;\sigma_{2},\;\mathrm{and}\;\sigma_{3}$ are the principal stresses of the stress tensor; and $\sigma_{xx},\;\sigma_{yy},\;\sigma_{zz},\;\sigma_{xy},\;\sigma_{yz},\;\mathrm{and}\;\sigma_{zx}$ are the normal and shear components of the stress tensor.

Burzyński understood that many materials are stronger in compressive load states rather than a tensile ones, so he proposed an improvement to the generalised Huber–Mises–Hencky hypothesis in the form below:

(46) $$\sigma_{B}=\frac{1}{2\varkappa }\left(1-\varkappa \right)\left(\sigma_{xx}+\sigma_{yy}+\sigma_{zz}\right)+\frac{1}{2\varkappa }[{\left(\varkappa -1\right)}^{2}{\left(\sigma_{xx}+\sigma_{yy}+\sigma_{zz}\right)}^{2}+\;+4\varkappa [\sigma_{xx}^{2}+\sigma_{yy}^{2}+\sigma_{zz}^{2}-\sigma_{xx}\sigma_{yy}-\sigma_{yy}\sigma_{zz}-\sigma_{zz}\sigma_{xx}+3\left(\sigma_{xy}^{2}+\sigma_{yz}^{2}+\sigma_{zx}^{2}\right)]{]}^{0.5}$$

or in a shorter form:

(47) $$\sigma_{B}=\frac{1}{2\varkappa }\left[3\left(\varkappa -1\right)\sigma_{m}+\sqrt{9{\left(\varkappa -1\right)}^{2}\sigma_{m}^{2}+4\varkappa \sigma_{HMH}^{2}}\;\right]$$

where $\sigma_{m}\;$is the mean normal stress and ϰ is the coefficient of asymmetry of the elastic area, which are determined by the following relationships:

(48) $$\sigma_{m}=\frac{1}{3}\left(\sigma_{xx}+\sigma_{yy}+\sigma_{zz}\right)$$

(49) $$\varkappa =\frac{k_{c}}{k_{t}}\equiv \frac{\sigma_{e}^{c}}{\sigma_{e}^{t}}$$

where, in turn: $\sigma_{e}^{t}\equiv k_{t}$ is the tensile yield limit, and $\sigma_{e}^{c}\equiv k_{c}$ is the compression yield limit. These parameters ought to be figured out during static compression and tension tests.

Note that some elements of this model have recently been developed in the literature, as in Banaś and Badur [8], where a numerical tool was prepared to extent the HMH surface modelling (of a cylinder) into the Burzyński surface (a paraboloid). The results of some numerical simulations alongside real experimental data have been examined in the available papers [15,28,30,31,32,33].

## 4. Experimental Procedure

**Figure 1:**
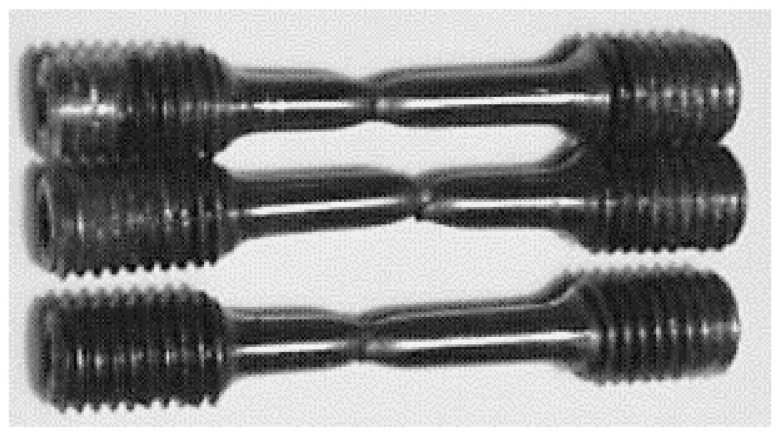
Ruptured samples after the tensile limit test (St12T steel, temp. 400 °C).

To verify the Burzyński effort hypothesis and evaluate the load asymmetry coefficient (Equation (50)), experimental measurements were obtained for the tensile and compressive limits. That approach is an improvement of the classic Huber–Mises–Hencky hypothesis, which assumes an equal limit in the tensile and compressive load regimes. Figure 1 presents a set of ruptured samples made of St12T steel after the tensile limit test at 400 °C. The diameter and length are 5 mm and 52 mm, respectively (excluding threads).

**Figure 2:**
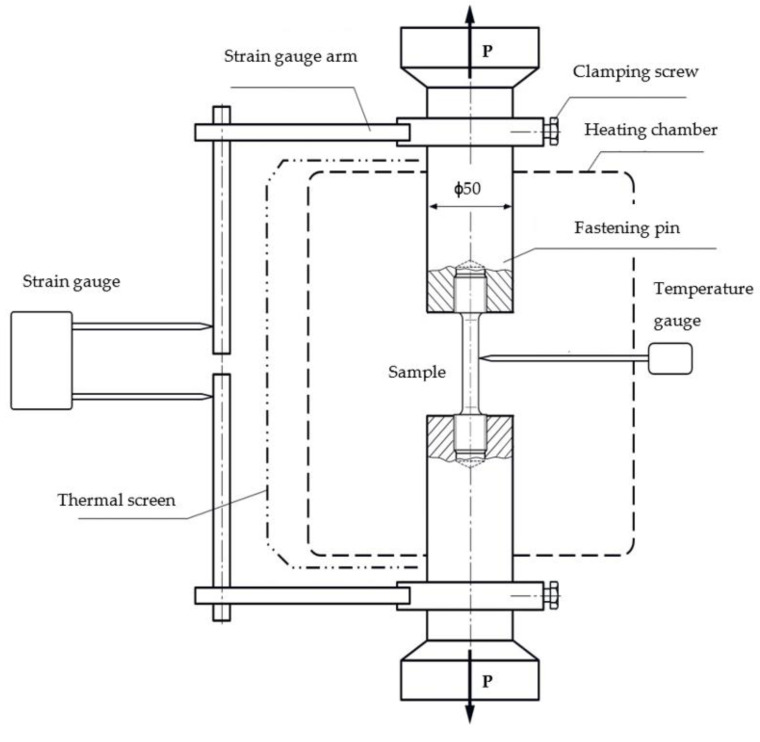
Experimental setup used for tensile and compressive limit tests.

The experiment is based on the Heckert EUS-20 hydraulic universal testing machine shown in Figure 2. A test performed at an elevated temperature was undertaken with a 20 kN load and an accuracy of ±0.05 kN. The heating system contains the heating chamber that is powered by the transformer, and equipped with a double-digit temperature control system (PT-0102 NVO Termoprylad) and temperature gauges. The temperature gauge accuracy is equal to ±1 °C. The strain gauge (MTS 634 11F-24) has a measurement capability between −2.5 mm and 5 mm, and its measurement class is equal to 0.5 (ISO 9513). The conversion from an analogue to digital signal was achieved using the L-Card E440 converter coupled with the Power Graph 3.3.8 software for storing and analysing data.

The experimental procedure is as follows:

(a) Screw-in the sample inside the fastening pins.
(b) Put the fastening pins inside the mount of the testing machine.
(c) Assure that the tensile force gauge shows zero.
(d) Close the heating chamber.
(e) Insert the temperature gauge into the heating chamber.
(f) Attach the thermal screen.
(g) Set the desired temperature (with variation equal to ±5 °C).
(h) Preload the sample with a force no higher than 200 N, check the position of the strain gauge arm, and attach the strain gauge.
(i) After 10 min of heating at a constant temperature, the load starts to grow at a maximum rate of 200 N/mm^2^ min.
(j) Once well inside the plastic stage, deformation rate is steadily increased up to 0.1 min^−1^ until rupture.
(k) The last step involves switching off heating, and performing data post-processing to evaluate yield strength $\sigma_{0.2}^{t}$.

## 5. Limit Properties of the St12T Steel

**Figure 3:**
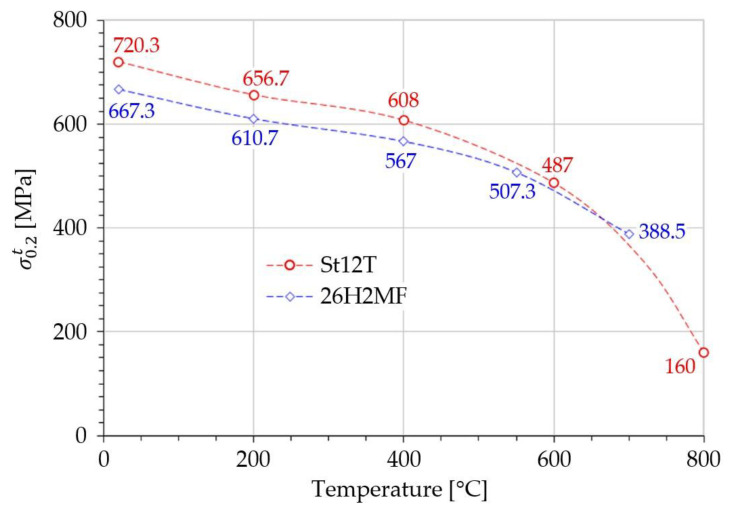
Tension elastic limits for two key heat-resistant steels (St12T and 26H2MF) at elevated temperatures (26H2MF curve added only for reference).

The most popular heat-resistant material used in power plants is St12T steel [4,8,14,15,16,28]. As mentioned in the previous section, static tensile and compressive tests were made to figure out the steel limit properties that are required for further calculations. To account for experimental variability three points were tested at a single temperature (Table 1). The only exception is the 800 °C case, where a single test was completed. Table 1 contains averaged values for each test condition:

Every model predicting the load and effort of power plant components ought to contain certain material properties that are dependent on temperature: Young’s modulus E=E(T); yield limit $\sigma_{e}=\sigma_{e}\left(T\right)$ or offset yield limit $\sigma_{0.2}=\sigma_{0.2}\left(T\right)$ (see Figure 3); and tensile strength $R_{m}=R_{m}\left(T\right)$. From the perspective of the Burzyński hypothesis, the impact of temperature on the elastic region coefficient of asymmetry (introduced by Burzyński) should also be taken into account according to the equation below:

(50) $$\varkappa =\varkappa \left(T\right)=\frac{\sigma_{e}^{c}\left(T\right)}{\sigma_{e}^{t}\left(T\right)}$$

**Figure 4:**
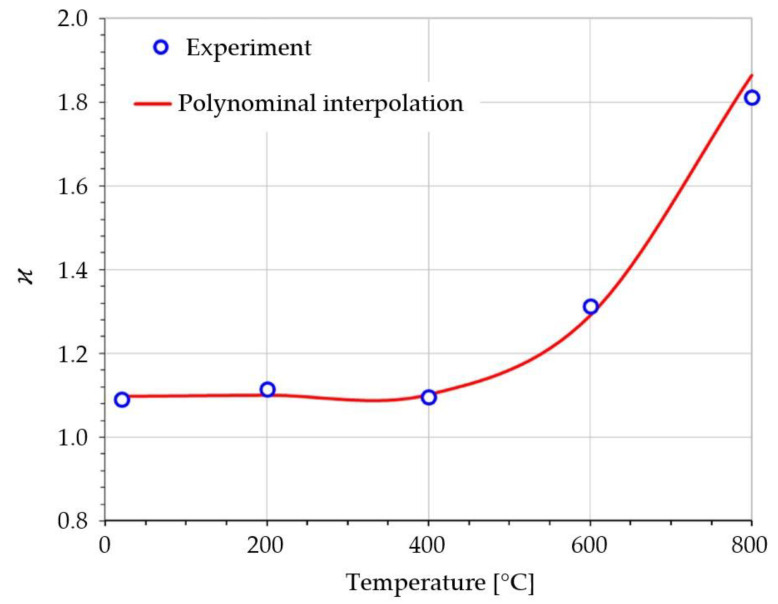
St12T steel elastic region asymmetry coefficient vs. temperature [30].

Elastic region asymmetry coefficients presented in Figure 4 were calculated according to Equation (50) and the yield strength limits presented in Table 1.

The polynomial interpolation curve presented on Figure 4 is described by Equation (51) and is valid for the temperature range of 20 °C–800 °C. This is a convenient form of the variable input parameter that is accepted by numerical analysis software.

(51) $$\varkappa =\varkappa \left(T\right)=4.1\cdot 10^{-9}\;T^{3}-2.57\cdot 10^{-6}\;T^{2}+4\cdot 10^{-4}\;T+1.09$$

In the following sections, the method based on the measured elastic region asymmetry coefficient (Equation (50)) will be called the vB (validated Burzyński) method.

## 6. Thermal Shifts of Burzyński Plastic Regions

The Burzyński hypothesis formulates plasticity as a function of three principal stresses $\left(\sigma_{1},\sigma_{2},\sigma_{3}\right)$ and is described by the following formula:

(52) $$\sigma_{1}^{2}+\sigma_{2}^{2}+\sigma_{3}^{2}-2\tilde{\nu }\left(\sigma_{1}\sigma_{2}+\sigma_{2}\sigma_{3}+\sigma_{3}\sigma_{1}\right)+\;+\left[\varkappa \left(T\right)-1\right]\sigma_{e}^{t}\left(T\right)\cdot \left(\sigma_{1}+\sigma_{2}+\sigma_{3}\right)-\varkappa \left(T\right)\cdot {\left[\sigma_{e}^{t}\left(T\right)\right]}^{2}=0$$

where: $\tilde{\nu }=\frac{\sigma_{e}^{c}\sigma_{e}^{t}}{2{\left(\sigma_{e}^{s}\right)}^{2}}-1$ is a coefficient of plasticity, and $\sigma_{e}^{s}$ denotes torsional yield limit. In the general case, the coefficient $\tilde{\nu }$ should also be modified to account for temperature dependency. However, $\tilde{\nu }$ is assumed to be 0.5 to govern the plasticity of St12T steel. The reason for this is the lack of measurements of torsional yield limit within the required temperature range. The same simplification of $\tilde{\nu }$ was adopted by Burzyński for brittle and plastic materials.

**Figure 5:**
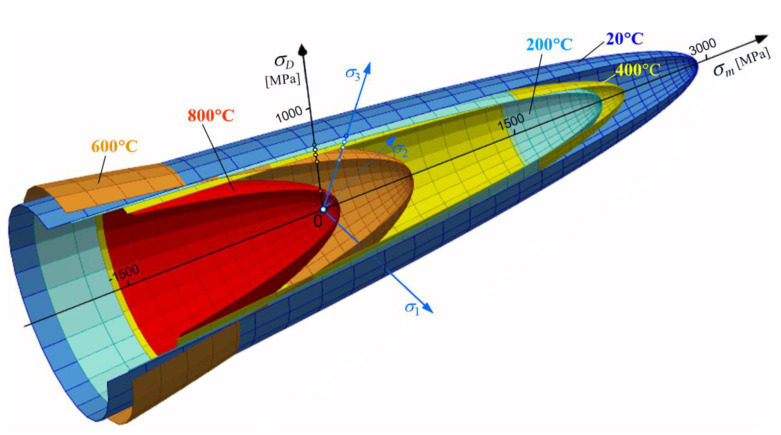
St12T plastic region paraboloid distribution, created using the Burzyński approach [30].

**Figure 6:**
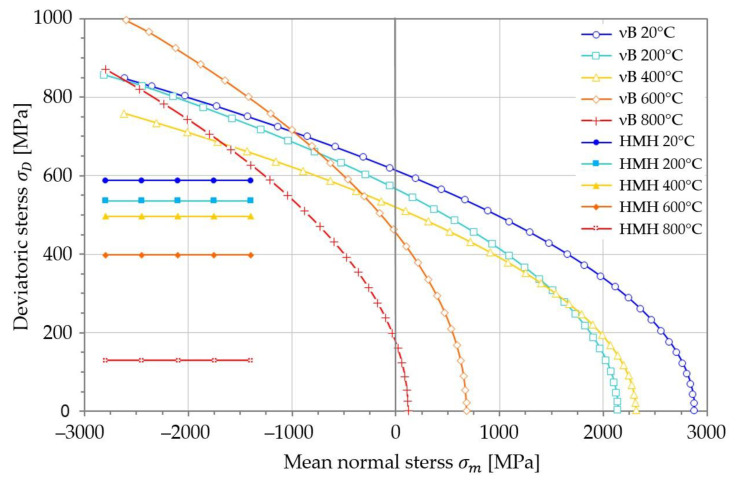
Comparison between HMH hypothesis and validated Burzyński hypothesis (vB) for St12T steel including a set of elevated temperatures [30].

The plastic limits governed by Equation (52) are plotted in Figure 5. Each surface accounts for a temperature (Table 1), a cycle asymmetry coefficient, and a tensile yield (elastic) offset [17]. The intersection curves between paraboloids and the reference plane that contains the deviatoric stress axis $\sigma_{D}$ (Figure 5) and the hydrostatic axis $\sigma_{m}$ ($\sigma_{1}=\sigma_{2}=\sigma_{3}$) are presented in Figure 6.

Figure 5 and Figure 6 are based on the average normal stress $\sigma_{m}$ at the hydrostatic axis, and the deviatoric stress $\sigma_{D}$ on the deviator stress axis. A 3D type of chart is required to mark the dependency between principal stresses and the deviatoric/hydrostatic axes.

The paraboloid shape of the tensile strength surfaces on Figure 5, and their cross-section on Figure 6 reveal the dependency between temperature and the plastic evolution of St12T steel (especially for regions of tension $\sigma_{1}+\sigma_{2}+\sigma_{3}>0$). The asymmetry coefficient ϰ clearly controls the shape and position of critical surfaces. It is very clear around 400 °C, where peak $\sigma_{m}$ stress is higher than at 200 °C (Figure 5). That inversion was observed for the first time in the literature. Additionally, Figure 3 proves that a plastic region also occurs at low temperatures (below 200 °C).

## 7. Conclusions

The Burzyński thermal effort hypothesis was created to capture the complex cycle-plastic behaviour of thermally loaded materials. In the presented paper, experimental confirmation was obtained for a heat-resistant St12T steel. The quasi-static load was applied at several thermal conditions to capture the heat-resistant steel parameters for the 20–800 °C range. As a result, it was found that yield limit values do not drop proportionally with an increase in temperature. Since both compressive and tensile stresses act during every thermal loading of a structure, important changes between the Huber–Mises–Hencky and Burzyński methodologies can be captured.

The evolution of the plastic deformation in St12T steel (Figure 6) clearly captures the impact of temperature on yield strength. That is particularly strong in the region of tension ($\sigma_{1}+\sigma_{2}+\sigma_{3}>0$). Temperature equal to 800 °C can be assumed as critical for tension, as the tip of that paraboloid (Figure 5) is close to zero at hydrostatic axis.

In summary, the authors have proved the advantage of the Burzyński three-parameter material effort model over the Huber–Mises–Hencky single-parameter approach. As a result, its use is recommended in industrial applications because it properly captures the interaction between temperature and yield surface position. One particularly valuable outcome is the strict mathematical description of the dependency between the temperature, shape, and position of the yield surface, which can be used in numerical simulations.

## Figures and Tables

**Table 1:** St12T steel mechanical properties [30].

Temperature	E(T) [GPa]	$\sigma_{0.2}^{t}(T)\;[\mathrm{MPa}]$	$\sigma_{0.2}^{c}(T)\;[\mathrm{MPa}]$	R_m_(T) [MPa]
20 °C	217.9	720.3	786.0	874.3
200 °C	206.9	656.7	731.7	804.3
400 °C	193.1	608.0	666.3	728.5
600 °C	141.8	487.0	639.3	570.5
800 °C	81.3	160.0	290.0	190

## Data Availability

The data presented in this study are available on request from the corresponding author. The data are not publicly available due to project restrictions.
